# Effects of elevated atmospheric [CO_2_] on grain starch characteristics in different specialized wheat

**DOI:** 10.3389/fpls.2023.1334053

**Published:** 2024-01-18

**Authors:** Qiongru Wei, Huqiang Pan, Yuxiu Yang, Shichao Tan, Liang Zheng, Huali Wang, Jie Zhang, Zhiyong Zhang, Yihao Wei, Xiaochun Wang, Xinming Ma, Shuping Xiong

**Affiliations:** ^1^ Collaborative Innovation Center of Henan Grain Crops, College of Agronomy, Henan Agricultural University, Zhengzhou, China; ^2^ College of Life Science, Henan Agricultural University, Zhengzhou, China

**Keywords:** elevated [CO_2_], specialized wheat, starch composition, starch physiological properties, gene expression

## Abstract

The increasing atmospheric [CO_2_] poses great challenges to wheat production. Currently, the response of starch characteristics in different specialized wheat cultivars to elevated [CO_2_], as well as the underlying physiological and molecular mechanisms remains unclear. Therefore, an experiment was conducted with open-top chambers to study the effects of ambient [CO_2_] [a(CO_2_)] and elevated [CO_2_] [e(CO_2_)] on photosynthetic performance, yield and starch characteristics of bread wheat (Zhengmai 369, ZM369) and biscuit wheat (Yangmai 15, YM15) from 2020 to 2022. The results demonstrated a significant improvement in photosynthetic performance, yield, amylose and amylopectin content, volume ratio of large granules under e[CO_2_]. Moreover, e[CO_2_] upregulated the gene expression and enzyme activities of *GBSS* (Granule-bound starch synthase) and *SSS* (Soluble starch synthase), increased starch pasting viscosity, gelatinization enthalpy and crystallinity. Compared to YM15, ZM369 exhibited a higher upregulation of *GBSSI*, greater increase in amylose content and volume ratio of large granules, as well as higher gelatinization enthalpy and crystallinity. However, ZM369 showed a lower increase in amylopectin content and a lower upregulation of *SSSI* and *SSSII*. Correlation analysis revealed amylose and amylopectin content had a positive correlation with GBSS and SSS, respectively, a significant positively correlation among the amylose and amylopectin content, starch granule volume, and pasting properties. In conclusion, these changes may enhance the utilization value of biscuit wheat but exhibit an opposite effect on bread wheat. The results provide a basis for selecting suitable wheat cultivars and ensuring food security under future climate change conditions.

## 1 Introduction

As a significant contributor to greenhouse gas, atmospheric concentration of CO_2_ has risen from original level of 280 µmol·mol^-1^ to approximately 417 µmol·mol^-1^ at present, which are concerned by the whole world (Sathee et al., 2022). Even with further emission reduction measures, it is estimated that global atmospheric CO_2_ concentration will reach around 600 µmol·mol^-1^ by the year 2050 (IPCC, 2021). Many journals, including *Nature*, have consistently published articles highlighting the significant effects of elevated [CO_2_] on both the yield and nutritional value of major crops (Mayer et al., 2014; Smith and Myers, 2018).

Wheat is a widely cultivated and highly adaptable crop (Weichert et al., 2017), and there are approximately 2.8 billion people worldwide who are dependent on wheat for their primary source of sustenance (Fahad et al., 2019). According to previous studies, elevated [CO_2_] enhanced photosynthetic rate of wheat (Bloom, 2014; Yadav et al., 2020), increasing photosynthetic assimilations, and improving biomass and yield (Ziska, 2022). Although elevated [CO_2_] promotes wheat yield, a certain amount of grain protein is diluted by the additional biomass, resulting in the “biomass dilution” effect. Consequently, it leads to decreases in nitrogen content and grain protein concentrations of wheat plants, which affects wheat quality (Bloom, 2014; Yadav et al., 2020). Wheat has specialized characteristics and can be classified into three specialized types according to different uses, namely strong gluten wheat, medium gluten wheat, and weak gluten wheat. According to Khalid et al. (2023), strong gluten wheat, also known as bread wheat, has a high protein content and strong gluten, attributing it suitable to make bread and noodles. On the other hand, weak gluten wheat, also known as biscuit wheat, has a low protein content and weak gluten, attributing it suitable to make cookies and pastries. Nevertheless, previous studies investigating the effects of elevated [CO_2_] on wheat quality did not consider the trait of specialized wheat, knowledge of elevated [CO_2_] on starch characteristics of bread wheat and biscuit wheat remain largely unknown.

Previous studies have indicated that elevated [CO_2_] increased starch content (Hogy and Fangmeier, 2008; Broberg et al., 2017). In a recent study, Jiang et al. (2021) observed multigenerational exposure to elevated [CO_2_] could significantly increase starch content in wheat grains, with amylose content increased by 3.5%, amylopectin content increased by 0.4% and amylose/amylopectin ratio increased by 3.12%. The amylose and amylopectin content are related to the heating time and stability of dough during the process of food baking (Yao et al., 2020). Dough with high amylose content and amylose/amylopectin ratio results in higher pasting temperatures, higher starch relative crystallinity (RC) and setback values (SB). Consequently, the dough tends to get hard and stale, leading to poor texture and storage properties (Nivelle et al., 2020), while higher amylose content has no significant effect on biscuit hardness or storage ability (Zhang et al., 2020). It has been reported that the increase of [CO_2_] decreased grain end-use quality, which was consist with changes caused by amylose and amylopectin content (Wroblewitz et al., 2014). Additionally, research conducted by An et al. (2021) showed that higher amylopectin content in bread resulted in better flavor scores due to its ability to form gel structures during heating, leading to larger volume and softer texture.

Bread wheat contains a higher volume of small granules (B-starch, diameter < 9.8 μm), conversely, in biscuit wheat, large granules (A-starch, diameter > 9.8 μm) dominate the most volume (Shevkani et al., 2017; Shang et al., 2020). Li et al. (2019) reported that elevated [CO_2_] significantly increased the proportion of large starch granules, but amylose and amylopectin content were not affected by elevated [CO_2_] in F1 and F4 wheat plants. The size of starch granules affects pasting characteristics, which are of great significance for starch processing quality. It is widely believed that exposure to elevated [CO_2_] results in significantly increasement in peak viscosity (PV), final viscosity (FV) and other important gelatinization parameters (Panozzo et al., 2014), thus rendering it more susceptible to gelatinization and improving its palatability (Jing et al., 2016). Relevant experiments conducted on rice have shown that elevated [CO_2_] enhanced the activity of GBSS and SSS, thereby promoting the synthesis of amylose and amylopectin (Xie et al., 2016; Jing et al., 2020). However, few research have focused on the mechanism underlying the changes of amylose and amylopectin content in different quality type of wheat. We hypothesize that e[CO_2_] changed amylose and amylopectin content by upregulation of genes involved in starch synthesis, moreover, starch characteristic of bread wheat and biscuit wheat response differently to e[CO_2_].

Currently, there is limited data available on the ratio of amylose/amylopectin, distribution of starch granule sizes and pasting properties in grains of different specialized wheat cultivars under elevated [CO_2_]. Additionally, the reasons for the difference of wheat starch component, starch granule sizes and pasting properties between cultivars remain unclear. Therefore, we investigated and analyzed the effects of e[CO_2_] on the yield, physiological indicators and starch characteristics of two specialized cultivars. In this study, the objectives were to: 1) analyze the effects of elevated [CO_2_] on yield, physiological traits, and starch characteristics of two specialized wheat cultivars; 2) explore the physiological and molecular mechanisms underlying the changes in starch content and quality induced by elevated [CO_2_] in specialized wheat.

## 2 Materials and methods

### 2.1 Experimental set-up

The experiment was conducted with two open-top chambers in Xuchang campus of the Henan Agricultural University (Henan, 113°48′34″ E, 34°7′56″ N, altitude 52.6 m), during two wheat seasons from 2020 to 2022. The experimental site is in the central of Huanghuaihai region. The meteorological conditions of the two seasons are shown in **Supplementary Figure 1**.

The experiment consisted of two specialized wheat cultivars and two atmospheric CO_2_ concentrations. The wheat cultivars selected for the experiment were Zhengmai 369 (ZM369) and Yangmai 15 (YM15). ZM369 is a bread wheat cultivar bred by the Wheat Research Institute of Henan Academy of Agricultural Sciences. It is a semi-winter wheat cultivar, with a growth period of 229 days. YM15 is a biscuit wheat cultivar bred by the Yangzhou Academy of Agricultural Sciences. It is a spring wheat cultivar with a growth period of 206 days.

The two CO_2_ concentration treatments were the ambient CO_2_ concentration (a[CO_2_], approximately 400 ppm) and elevated CO_2_ concentration (e[CO_2_], approximately 600 ppm). Elevated CO_2_ concentration was performed using open-top chamber (Johnson et al., 2013). Each chamber is 5.6 meters in length, 3.9 meters in width, and 1.93 meters in height, with an area of 21.84 m^2^. Sensors were installed inside the chambers to monitor real-time CO_2_ concentration data during the experiment (**Supplementary Figure 1**). The CO_2_ gas transporting pipe was raised continuously throughout the wheat growing season to maintain a height of about 10 cm above the wheat canopy. The CO_2_ concentration in e[CO_2_] chamber was controlled by using the pressure-reducing valve (Shanghai Tianchuan Instrument), the CO_2_ concentration control system is shown in the **Supplementary Figure 2**. Elevated CO_2_ concentration was treated from wheat reviving stage to maturity, on 23 February 2021, and 25 February 2022, respectively.

The wheat was grown in pots (32 cm in height, 35 cm in diameter), with 15 kg soil and the soil depth was 30 cm. The soil used for potting is sandy loam soil, all pots are buried in the soil. The soil contained 1.30 g total N kg^-1^, 50.15 mg available N kg^-1^, 8.54 mg available phosphorus of kg^-1^, 235.14 mg available potassium of kg^-1^, and 16.2 g organic matter kg^-1^. Before sowing, each pot was fertilized with 5.94 g pure sodium nitrate (equivalent to 0.98 g pure nitrogen), 8.7 g superphosphate (containing 1.044 g P_2_O_5_), and 1.74 g potassium chloride (containing 1.044 g K_2_O). At the jointing stage, each pot was additionally fertilized with 5.94 g sodium nitrate. The wheat of two seasons were sowed on October 20, 2020, and October 22, 2021, respectively. Sow two wheat cultivars in each chamber at the same time, 30 pots of each cultivar, for a total of 60 pots. with 10 seeds per pot.

### 2.2 Determination indicators and methods

#### 2.2.1 Photosynthetic properties and product accumulation

Marked spikes that bloomed on the same day. At the flowering stage and post-anthesis (Days After Pollination (DAP)), including 8DAP, 16DAP, 24DAP, 10 flag leaves were collected from different pots, measured net photosynthetic rate (Pn) by Li-6400XT portable photosynthetic detector (Li-COR, Lincoln, NE, USA) from 9:00 am to 11:30 am. In addition, measured the chlorophyll content of flag leaves by a the SPAD-502 (Konica Minolta, Japan) at Jointing Stage, Flowering Stage, 8DAP, 16DAP and 24DAP.

The plant samples obtained in Flowering Stage, 8DAP, 16DAP, 24DAP and 32 DAP were dried at 105°C for 30 min, then 80°C for 8 hours, weighed and recorded. At each period, three wheat plants were selected from different pots, and the sampling was repeated three times. At maturity, counted the number of spikes per pot and grains per spike, after harvest, air-dried to a moisture content of about 13%. Then weighted the yield of per pot and 1000-grain. All samples had three biological replicates.

#### 2.2.2 Sucrose, starch and protein content in grains

After drying the spikes harvested at different periods in 2.2.1, and the kernels were ground into powder with a grinder. The sucrose content was determined according to the anthrone method (Yemm and Willis, 1954). The residue obtained after ethanol extraction was collected and the starch in it was converted to sucrose using a perchloric acid hydrolysis method (Wang and Copeland, 2015). After measuring the sucrose content using the anthrone method, starch content was sucrose content multiplied by 0.9.

The plant materials were digested by the method of Eastin (1978), 0.1 g ground grains and 5 ml of concentrated sulfuric acid were added into a 50 ml large test tube, and the volume was adjusted to 50 ml after digestion in an elimination furnace at 380°C. After dilution four times, the total nitrogen content was determined by a SEAL AutoAnalyzer 3 continuous flow analyzer (Bran+Luebbe GmbH company, Norderstedt, Germany). The protein content was total nitrogen content multiplied by 5.7.

The mature grains were air-dried and ground into flour using a German Brabender small test mill. The total starch content was determined referring to the method of HE (1985), the amylose and amylopectin content were determined according to the two-wavelength method proposed by (Zhu et al., 2008), and the determination wavelength and reference wavelength of amylose were 630 nm and 446 nm respectively, and amylopectin were 540 nm and 750 nm, respectively.

#### 2.2.3 Indicators of starch synthetase enzymes

Activity of starch synthetase enzymes. Fresh wheat spike samples at 16DAP were collected, and the grains were quickly frozen in liquid nitrogen and transferred to -80°C for storage. SPS (sucrose phosphate synthase) and SS (sucrose synthase) enzyme activities were determined according to Guy et al. (1992). GBSS and SSS enzyme activities were determined by method of Cheng (2001) and Nakamura et al. (1989), respectively.

Starch synthesis enzyme gene expression. Total RNA was extracted from wheat grains using a TransZol Plant kit (ET121) following the instructions. The first strand of cDNA was synthesized by a HiScript III RT SuperMix for qPCR (+gDNA wiper) kit (Vazyme, China). According to the *GBSS* and *SSS* cDNA gene sequences released by the National Center for BioInformation (NCBI) Technology, Primer 5.0 was used to design primers (**Table 1**). The specificity of Primer Blast in NCBI was evaluated by the homologous comparison function. The primers were synthesized by Henan Shangya Biotechnology Company. The cDNA samples were quantified by PCR with actin primers. The reaction was performed in a 96-well PCR plate using the Vazyme ChamQ Universal SYBR qPCR Master Mix kit. The reaction was amplified on the CFX96 RT-PCR instrument, and each sample was repeated three times. At the end of the cycle, the dissolution curve was analyzed at 60 °C–95 °C.

**Table 1:** Primer sequence used for analysis of target gene expression.

Gene name	Gene ID	Primer sequence (5’-3’)
* TaGBSSI*	AF286320	F: GAAGTATGGGTTGTTGTTGAGG R: CCTCAACAACAACCCATACTTC
* TaSSSI*	AJ292521	F: AAGGTGGACAGGGCCTCAAT R: CACTTGTCTGTGGTGGGGTTC
* TaSSSIIa*	AJ269503	F: GCTCCTTTGAACATCACCAGAACCA R: CCGCAACATCTCCAAGACCACCT
* TaSSSIIIa*	AF258608	F:CGGAACCGACCCAATCAA R:GCATCCTGCCTATCAACA
* TaActin*	AB181991	F: GTTCCAATCTATGAGGGATACACGC R: GAACCTCCACTGAGAACAACATTACC

#### 2.2.4 Grain starch characteristics

Starch morphology observation. The starch was separated by the dough method (Song et al., 2020). A total of 0.1 g starch and 5 mL distilled water were added into a 10 mL centrifugal tube. The mixture was shaken adequately and refrigerated at 4 °C. The 10 μL starch solution was absorbed by a pipettor and evenly dispersed on the conductive adhesive of the sample table. Observed the starch granule morphology using scanning electron microscope (Hitachi SU3500 SEM), and photographed at 1000× magnification.

Particle size distribution analysis. Suspended the starch sample in water, analyzed starch granules distribution by LS13320 (Beckman, USA). A total of 0.1 g starch and 5 mL distilled water were added into a 10 mL centrifuge tube, then the mixture was shaken and refrigerated at 4 °C for 1 h, and the mixture was shaken once every 10 minutes during the period. Then, the starch solution was transferred to the dispersion box of the laser diffraction particle size analyzer, and the distribution of wheat starch granules was determined.

X-ray diffraction analysis. Starch XRD and relative crystallinity were measured by method of Man (Man et al., 2014). The diffractometer operates at 200 mA and 40 kV. The diffraction Angle (2θ) has a scanning area ranging from 5° to 40°, a step size of 0.02°, and a counting time of 0.6 s. The X-ray diffractometer of Nippon Mini flex600 was used for XRD analysis. The relative crystallinity (RC, %) was calculated by MDI Jade 6.5 software.

Pasting characteristics. A rapid viscosity analyzer (RVA-4500, Perten, Sweden) was used in this experiment. According to the operation instructions, 3 g flour and 25 mL water were added into the mixing cylinder, and the samples were stirred until no dough was scattered. Every gelatinization parameter was detected under the control of the set program.

Thermal properties. A total of 5 mg (accurate to 0.01 mg) starch was put in an aluminum crucible (25 μL, D = 5 mm), and 10 ml water was poured. Samples were sealed, stood at 4°C for 24 h, and then heated in a DSC (differential scanning calorimeter, Natch, Germany). A closed empty aluminum crucible was used as a contrast to calibrate the DSC analyzer.

### 2.3 Statistical analysis

The average values were calculated using Microsoft Excel 365. The software Origin2021 pro and Adobe illustrator cc 2017 were used for mapping. SPSS 25.0 was used to performed ANOVA and correlation analyses (SPSS Inc, Chicago, USA). Furthermore, Duncan’s multiple comparisons were performed for the differences among the treatments.

## 3 Results

### 3.1 Effects of e[CO_2_] on chlorophyll content, photosynthetic rate and yield

**Figure 1:**
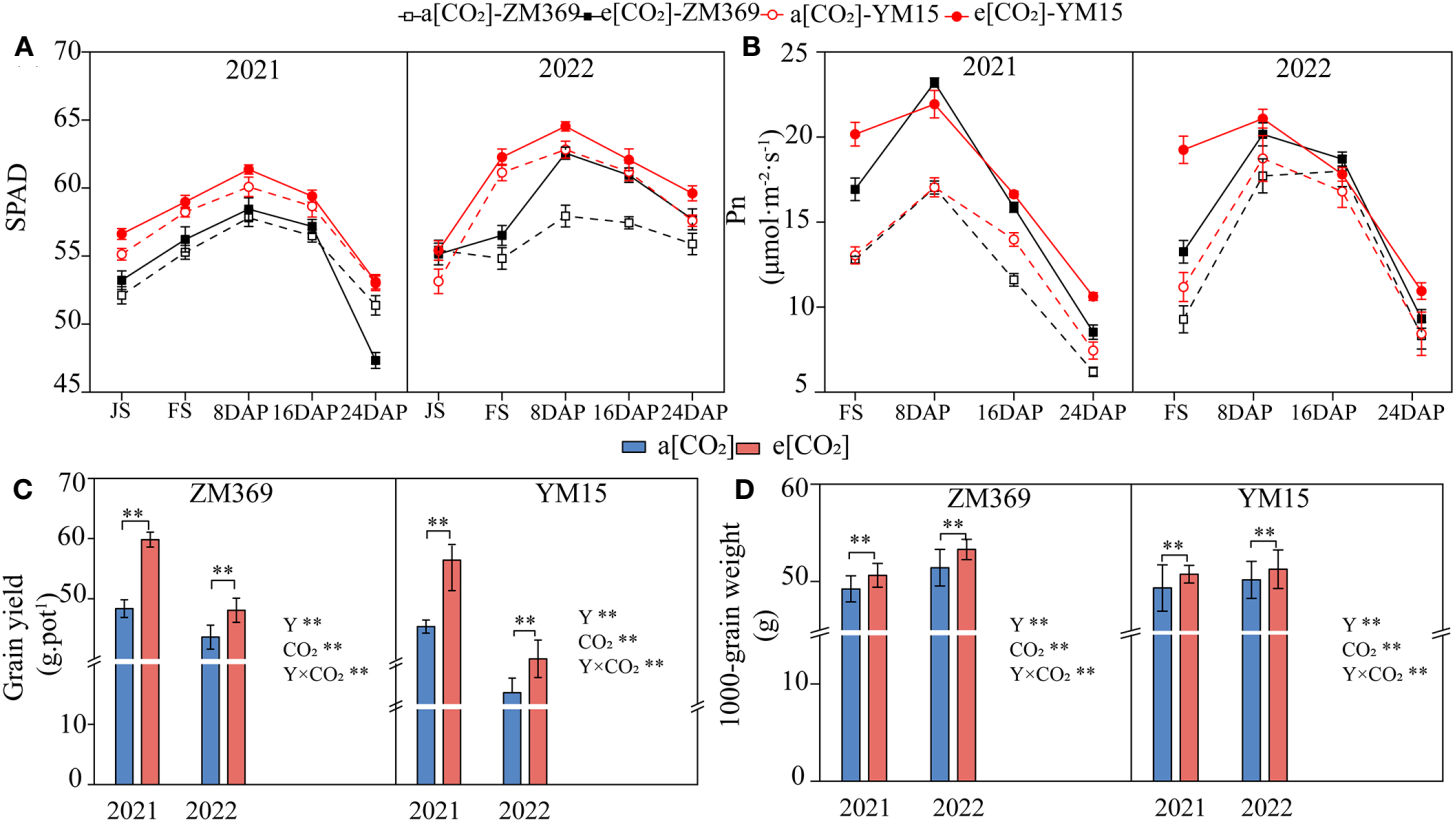
Effect of elevated [CO_2_] on photosynthetic performance and yield of two wheat cultivars. **(A)** SPAD value. **(B)** Net photosynthetic rate. **(C)** Grain yield weight per pot. **(D)** 1000-grain weight. JS, FS, 8 DAP, 16 DAP, 24 DAP and 32 DAP respectively represent Jointing Stage, Flowering Stage, 8, 16, 24 and 32 Days After Pollination (DAP). Results are presented as means ± standard deviation, and SD is indicated by error bars. Each mean has at least 3 biological replicates. Mean values were compared using the least significance difference test (LSD). Y, year; **, significant differences at *P* < 0.01.

Leaf chlorophyll content is closely related to the plant growth. The chlorophyll content (measured as SPAD value) increased first and subsequently decreased at grain filling stage. Compared with a[CO_2_], e[CO_2_] significantly increased the chlorophyll content in both cultivars, with ZM369 increasing by 0.81%-4.54%, while YM15 by 1.36%-3.49%. Interestingly, YM15 exhibited higher chlorophyll content compared to ZM369 under both a[CO_2_] and e[CO_2_] condition (**Figure 1A**). The chlorophyll content of both cultivars in 2022 (2021-2022) was higher than that in 2021 (2020-2021). The net photosynthetic rate of the two cultivars exhibited an initial increase followed by a gradual decrease, consistent with the changes in chlorophyll content, with the peak value appearing 8 days after flowering. Notably, e[CO_2_] significantly enhanced the net photosynthetic rate of both cultivars (**Figure 1B**).

The increase in chlorophyll content and photosynthetic rate provides energy ensuring substance accumulation during wheat growth, dry matter accumulation steadily increased during the growth period and peaked at maturity. Compared with a[CO_2_], e[CO_2_] significantly enhanced dry matter accumulation of two cultivars, with ZM369 increasing by 10.63%-41.91%, while YM15 by 8.45%-35.88% (**Supplementary Figure 3**). Compared with a[CO_2_], e[CO_2_] significantly improved the grain yield and thousand grain weight of two cultivars (**Figures 1C, D**). In 2021, the yield and thousand grain weight of ZM369 increased significantly by 23.72% and 2.83%, YM15 increased significantly by 17.59% and 2.86%, respectively. While in 2022, the yield and thousand grain weight of ZM369 increased significantly by 10.22% and 3.66%, the number of YM15 was 10.84% and 2.19%. The yield of both cultivars in 2022 was lower than in 2021. The two factors of [CO_2_], year and their interaction had significant effects on grain yield.

### 3.2 Effects of e[CO_2_] on sucrose and starch content

**Figure 2:**
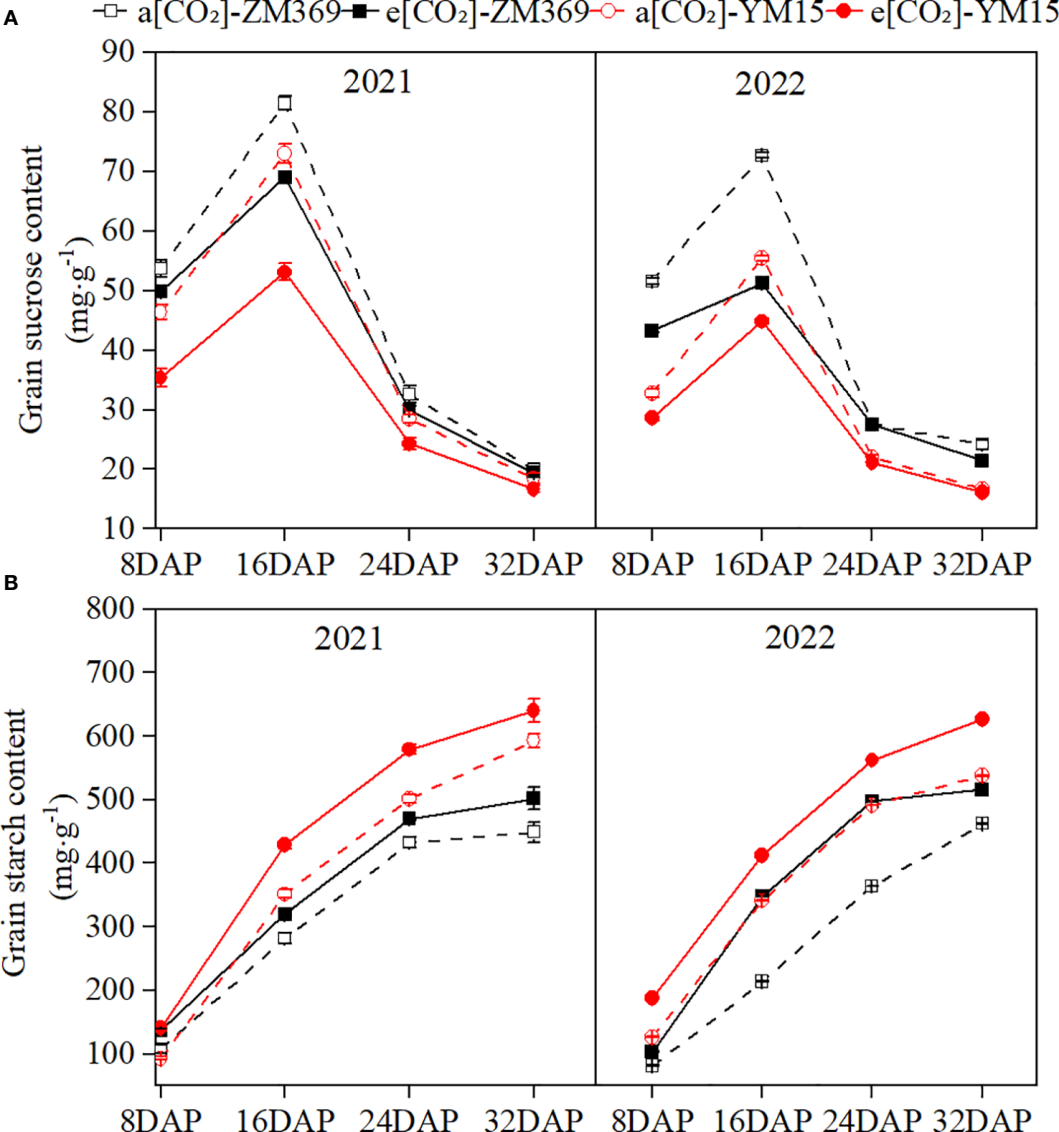
Effect of elevated [CO_2_] on sucrose and starch content of two wheat cultivars. **(A)** Starch content. **(B)** Sucrose content. Results are presented as means ± standard deviation, and SD is indicated by error bars. Each mean has at least 3 biological replicates.

Carbohydrate are the main products of plant photosynthesis, photoassimilate from the leaves are transported via the phloem to the grains during the grain filling stage, where starch was synthesized through enzymatic reactions. In this experiment, the sucrose content increased first and then decreased during the grain filling stage, reaching its peak at 16 DAP, which was consistent with the change of photosynthetic rate. The starch content continuously increased throughout the filling stage, reaching its highest at maturity. Compared to a[CO_2_], e[CO_2_] significantly decreased sucrose content and increased starch content in the grains of both cultivars (**Figures 2A, B**). YM15 had lower sucrose content but higher starch content than ZM369 in 2021 and 2022. This may be explained by starch synthesis uses sucrose as a substrate, leading to consumption of more sucrose in YM15 for starch synthesis, resulting in lower sucrose content but higher starch content than ZM369.

**Figure 3:**
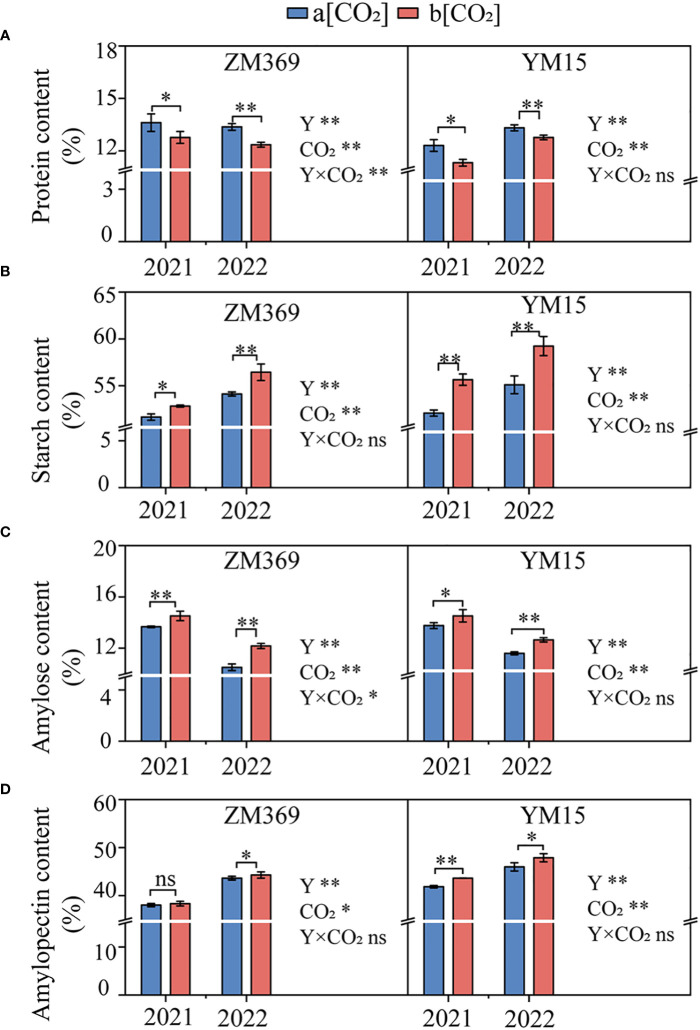
Effect of elevated [CO_2_] on protein and starch content of two wheat cultivars at maturity. **(A)** Protein content. **(B)** Starch content. **(C)** Amylose content. **(D)** Amylopectin content. Results are presented as means ± standard deviation, and SD is indicated by error bars. Each mean has at least 3 biological replicates. One-way analysis of variance (ANOVA) was used to test the significant differences between treatments, and different letters superscripts indicated significant differences (p<0.05). Mean values were compared using the least significance difference test (LSD). Y, Year; ns, non-significant; *, *P <*0.05, **, *P <*0.01.

The changes in grain total starch, amylose, amylopectin and protein contents of two specialized cultivars at maturity under e[CO_2_] are shown in **Figure 3**. The results indicate that e[CO_2_] significantly enhanced the total starch, amylose and amylopectin contents, as well as amylose/amylopectin ratio, while significantly decreasing protein content. The total starch content of ZM369 and YM15 increased by 3.28% and 4.69%, respectively. Likewise, the amylose content rose by 11.06% and 6.50%, the amylopectin content increased by 1.17% and 4.19%, as for amylose/amylopectin ratio, the numbers were 9.84% and 2.19%, respectively. However, the protein content in ZM369 and YM15 decreased by 7.44% and 8.53% (P<0.01), respectively. Moreover, the total starch and amylopectin content in YM15 were significantly higher than those in ZM369, while the amylose/amylopectin ratio and protein content of YM15 was significantly lower than that in ZM369. Additionally, ZM369 exhibited a greater increase in amylose content, whereas YM15 displayed a greater increase in amylopectin content.

### 3.3 Effects of e[CO_2_] on wheat starch structural properties

**Figure 4:**
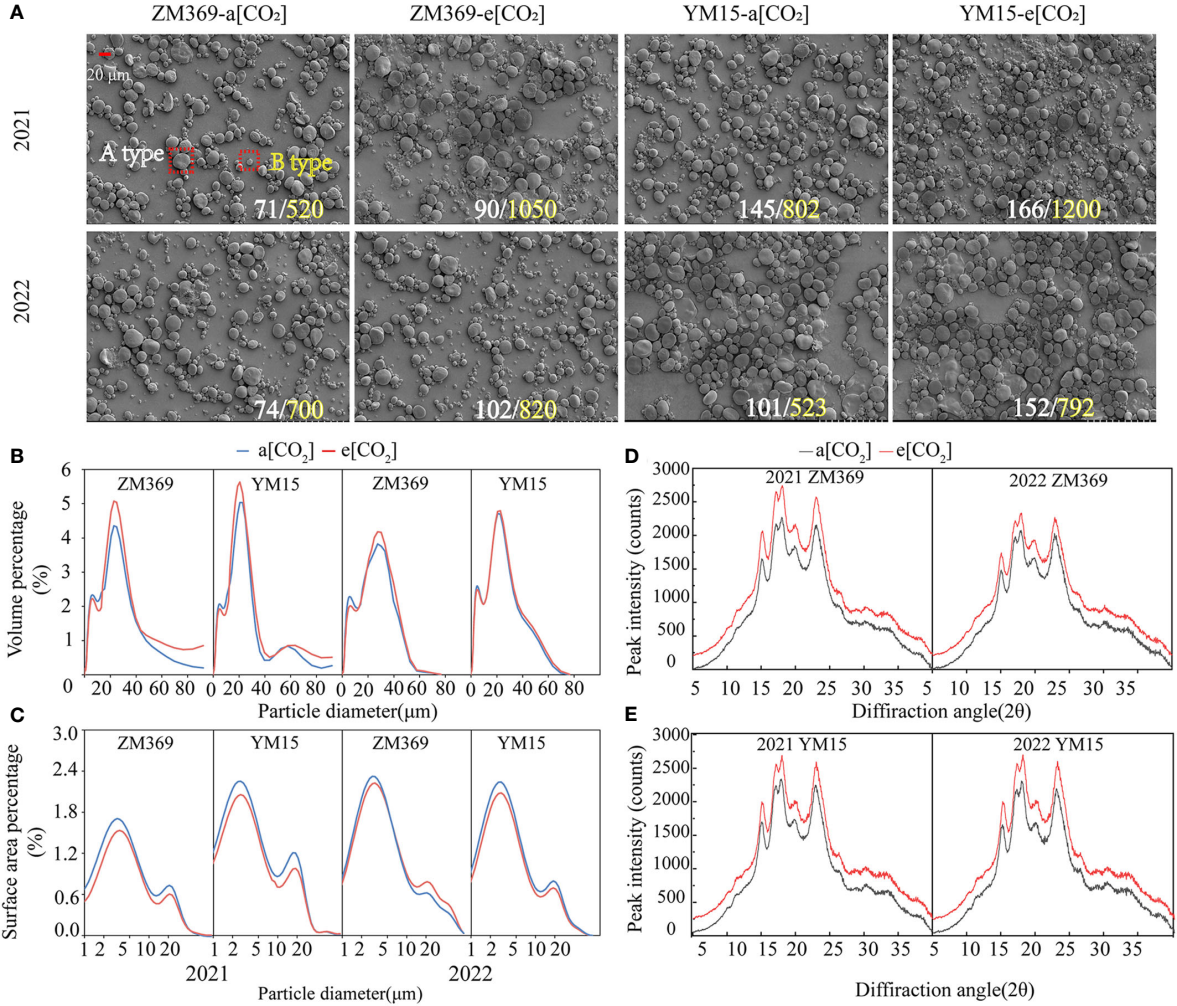
The starch granule morphology, volume, surface area percentage and crystalline structure analysis of wheat cultivar ZM369 and YM15 under a[CO_2_] and e[CO_2_] conditions. **(A)** Starch granule morphology on SEM. The white number represents the number of large granules, and the yellow number represents the number of small granules in the field of vision. **(B)** The volume percentage of two cultivars. **(C)** Peak intensity of wheat cultivars ZM369. **(D)** The surface area percentage of two cultivars. **(E)** Peak intensity of wheat cultivars YM15.

Starch exists in the form of starch granules in wheat endosperm. The amylose and amylopectin content increased by e[CO_2_], in order to explore whether e[CO_2_] changed starch morphological structure, scanning electron microscopy was used to examine starch morphology, particle size distribution. According to **Table 2**, more than 99.99% of starch particles were categorized as medium and small-sized, constituting the primary components of starch. Compared with a[CO_2_], the morphology of both large and small granules remained unchanged under e[CO_2_] (**Figure 4A**). However, analysis of the composition of different sized starch particles revealed that e[CO_2_] significantly increased the volume ratio of large granules and decreased the volume ratio of small granules. Moreover, e[CO_2_] resulted in reduction in the surface area percentage of large granules in both wheat cultivars (**Table 2**). ZM369 exhibited lower content of both large and small granules compared to YM15, as well as a lower ratio of large/small granules.

**Table 2:** The starch granule size distribution of cultivar ZM369 and YM15 under a[CO_2_] and e[CO_2_].

Years	Cultivars	Treatment	Volume percentage (%)		Surface area percentage (%)		Number percentage (%)	
			B (<10 μm)	A (>10 μm)	B (<10 μm)	A (>10 μm)	B (<10 μm)	A (>10 μm)
2021	ZM369	a[CO_2_]	39.1a	60.4c	89.9a	10.1a	99.9991ab	0.00087b
		e[CO_2_]	35.2b	64.4a	91.5a	8.4b	99.9993a	0.00065b
	YM15	a[CO_2_]	38.6ab	61.3bc	89.3a	10.7a	99.9989b	0.00121a
		e[CO_2_]	36.7bc	63.2ab	89.7a	10.3a	99.9989b	0.00113a
2022	ZM369	a[CO_2_]	37.9c	62.1b	87c	13a	99.9987a	0.00127a
		e[CO_2_]	32.6d	67.3a	89.3b	10.7b	99.9989a	0.00111a
	YM15	a[CO_2_]	45.5a	54.5d	89.6b	10.3b	99.9992a	0.00079b
		e[CO_2_]	42.4b	57.6c	91.8a	8.2c	99.9988a	0.00121a

Different lower-case letters in the same column indicate a significant difference in the same year (P < 0.05).

The starch granules exhibited bimodal curves in both volume and surface area distributions, covering a diameter range of 0.4 to 100 μm. Analysis of the volume distribution in 2021 and 2022 showed that the primary diffraction peaks of ZM369 were presented at 22.73 μm and 27.39 μm respectively, whereas for YM15 it was presented at 20.73 μm in both years (**Figure 4B**). Additionally, the surface area distribution indicated that the primary peak surface area for ZM369 was observed at 3.206 μm, while for YM15 it was at 2.66 μm (**Figure 4C**).

The X-ray diffraction patterns of starch granules were classified into three types: A-type, B-type and C-type. Both cultivars in this study exhibited an A-type pattern in the X-ray diffraction pattern analysis, with a unimodal structure at 15°, 20° and 23°, and a typical bimodal structure at 17° and 18°. The peak diffraction intensities in 2021 and 2022 were 18°>17°>23°>20°>15°. E[CO_2_] significantly increased the relative diffraction intensity at each diffraction peak for two cultivars (**Figures 4D, E**). Furthermore, the RC of starch in two cultivars significantly increased in 2021 and 2022 (**Table 3**). These results indicated that e[CO_2_] had a significant effect on the size distribution of starch granules.

**Table 3:** The starch pasting and thermal properties of cultivar ZM369 and YM15 under a[CO_2_] and e[CO_2_].

Year	Cultivars	Treatment	PV (cP)	TV (cP)	BD (cP)	FV (cP)	SB (cP)	To (°C)	Tp(°C)	Tc(°C)	ΔH(J/g)	RC (%)
2021	YM15	a[CO_2_]	1950b	1340bc	634a	2382bc	1042b	56.1c	62.2b	67.5a	10.1b	24.6d
		e[CO_2_]	2073a	1475a	598b	2549a	1073ab	57.6b	62.9b	64.9b	10.6b	27.7b
	ZM369	a[CO_2_]	1533d	1314c	205c	2347c	1053b	58.4b	64.6a	69.2a	11.7b	25.4c
		e[CO_2_]	1585c	1373b	224c	2438b	1100a	59.9a	65.3a	69.1a	16.2a	29.2a
2022	YM15	a[CO_2_]	1924b	1326a	706b	2270a	979a	57.9b	62.4b	66.8a	7.2d	24.4b
		e[CO_2_]	2446a	1330a	1105a	2435a	1073a	58.7b	62.8b	67.7a	9.7c	26.0a
	ZM369	a[CO_2_]	1465d	1059b	336d	2026b	986.7a	58.9b	65.1a	68.9a	13.6b	24.7b
		e[CO_2_]	1701c	1248a	523c	2303a	1034.7a	60.2a	65.3a	68.3a	15.8a	27.4a

Different lower-case letters in the same column indicate a significant difference in the same year (P < 0.05).

PV, peak viscosity; TV, through viscosity; BD, breakdown viscosity; FV, final viscosity; SB, setback viscosity; To, onset temperature; Tp, peak gelatinization temperature; Tc, conclusion temperature; ΔH, gelatinization enthalpy; RC, relative crystallinity.

### 3.4 Effects of e[CO_2_] on starch pasting and thermal properties

Changes in the amylose and amylopectin content, as well as starch granule size, can affect the pasting and thermal properties. **Table 3** shows the pasting and thermal properties of ZM369 and YM15 in response to different CO_2_ concentrations. Compared with a[CO_2_], e[CO_2_] significantly increased PV of ZM369 and YM15 by 16.65% and 9.57%, the trough viscosity (TV) increased by 5.17% and 10.51% for ZM369 and YM15, respectively. The FV increased by 8.42% for ZM369 and 7.16% for YM15. The breakdown value (BD) significantly increased by 27.18% for ZM369 and 38.08% for YM15, and the BD of ZM369 was significantly lower than YM15. The SB showed an increase under e[CO_2_] conditions, however, the increase magnitude varied between the two cultivars, the data of ZM369 in 2021 and YM15 in 2022 reached significant levels (**Table 3**). Analysis of variance showed significant differences in pasting properties between the two cultivars and two [CO_2_] treatments, while the interaction between cultivar and [CO_2_] had no significant impact on pasting properties.

Differential scanning calorimetry (DSC) can measure starch heat absorption and release during the gelatinization processes. This study found that the onset temperature (To), peak temperature (Tp), and gelatinization enthalpy (ΔH) increased under e[CO_2_] treatment in 2021 and 2022. YM15 exhibited significantly higher gelatinization enthalpy than ZM369 under e[CO_2_] in 2021 and 2022. Analysis of variance revealed significant differences in each parameter among the wheat cultivars, and the e[CO_2_] treatment had a significant impact on all parameters apart from Tc. The interaction between cultivar and [CO_2_] had no significant effect on the thermal properties.

### 3.5 Effects of e[CO_2_] on starch synthase activity and gene expression

**Figure 5:**
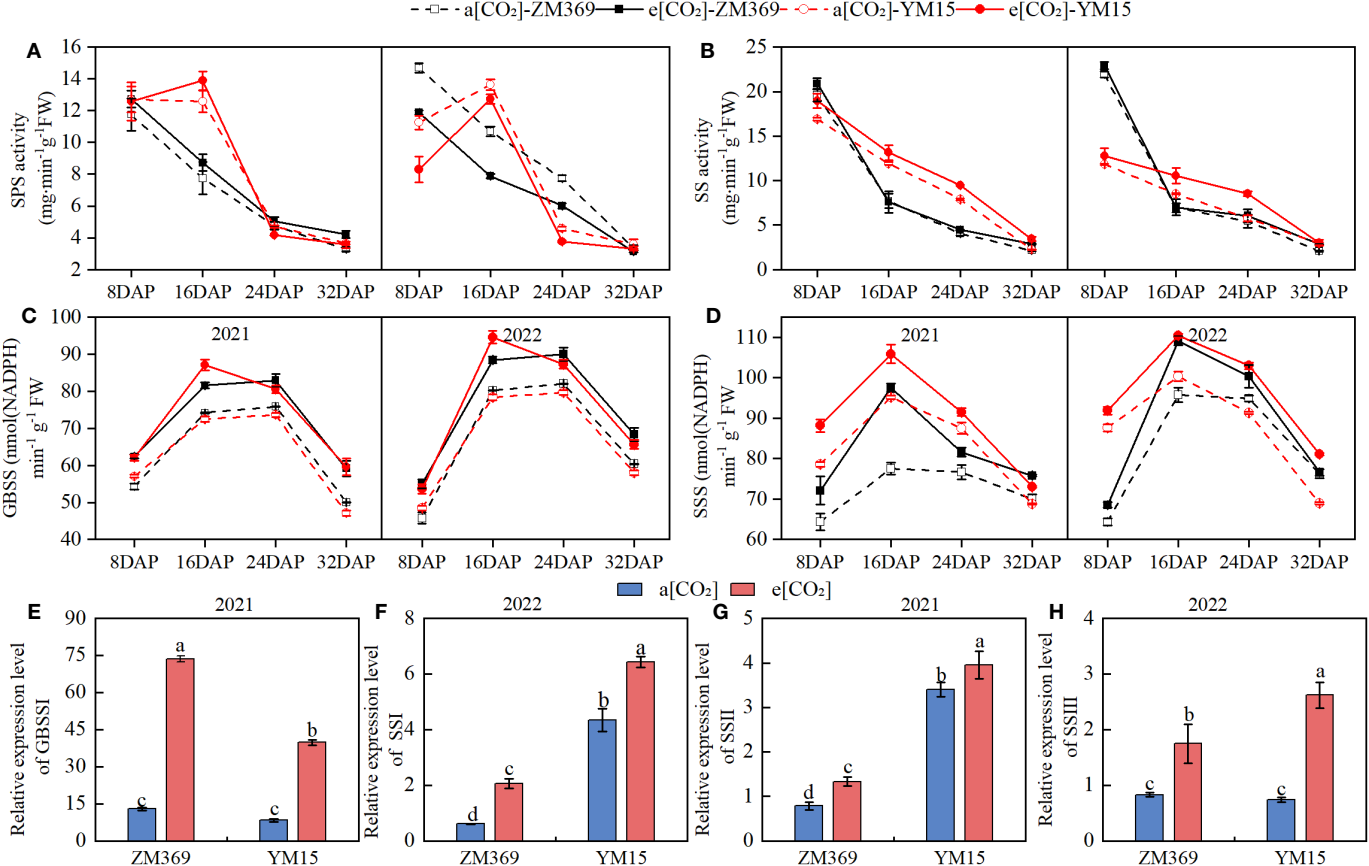
Effect of elevated [CO_2_] on enzyme activities and gene expression of two wheat cultivars. **(A)** SPS activities. **(B)** SS activities. **(C)** GBSS activities. **(D)** SSS activities. **(E)** Relative expression level of *GBSSI*. **(F)** Relative expression level of *SSSI*. **(G)** Relative expression level of *SSSII.* **(H)** Relative expression level of *SSSIII.* Results are presented as means ± standard deviation, and SD is indicated by error bars. Each mean has at least 3 biological replicates. One-way analysis of variance (ANOVA) was used to test the significant differences between treatments, and different letters superscripts indicated significant differences (*P <*0.05). Mean values were compared using the least significance difference test (LSD).

SPS and SS enzymes are responsible for sucrose synthesis and degradation. As shown in **Figure 5**, the activities of these enzymes in ZM369 gradually decrease during the growth, while the trend for YM15 is first increasing and then decreasing. Compared with a[CO_2_], e[CO_2_] increased the enzyme activities of SPS and SS of two cultivars. The activities of these two enzymes are related to the content and proportion of amylose and amylopectin in mature grain. In this study, the enzyme activities of GBSS and SSS showed the trend of initial increase followed by decrease in wheat. E[CO_2_] significantly increased the enzyme activities of SS and SSS in both cultivars compared with a[CO_2_]. When comparing the two cultivars, YM15 showed a greater increase of SSS activity, while ZM369 exhibited a larger increase of GBSS activity. The activities of both SS and SSS enzymes in YM15 grains were higher than those in ZM369 during the filling stage under e[CO_2_] treatment.

The starch content of grains significantly increased under the e[CO_2_] condition, to investigate the molecular mechanisms underlying the increase in starch content, we conducted qPCR analysis of the relative expression levels of *GBSSI*, *SSSI*, *SSSII*, and *SSSIII* genes. The results showed that compared with a[CO_2_], e[CO_2_] significantly upregulated the relative expression level of *GBSSI*, *SSSI*, *SSSII* and *SSSIII* in both cultivars. When comparing the two cultivars under e[CO_2_] conditions, it was found that the expression levels of *SSSI*, *SSSII* and *SSSIII* genes in YM15 were 3.14, 2.97, and 1.5 times that of ZM369. Moreover, the *GBSSI* gene expression level of ZM369 was 1.84 times higher than that in YM15.

### 3.6 Correlation analysis

**Figure 6:**
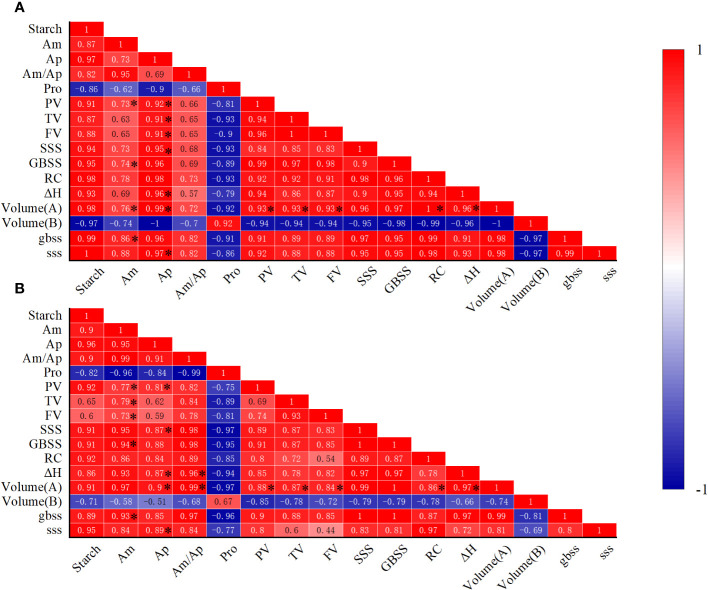
Pearson correlation matrices of physicochemical properties of wheat. **(A)** Pearson correlation in 2021. **(B)** Pearson correlation in 2022. Data with single asterisk (*) are statistically significant at P <0.05. Am, amylose content; Ap, amylopectin content; Pro, protein content; PV, peak viscosity; TV, trough viscosity; FV, final viscosity; SSS, enzyme activity of SSS; GBSS, enzyme activity of GBSS; ΔH, gelatinization enthalpy; RC, relative crystallinity; Volume **(A)**, volume ratio of large granule; Volume **(B)**, volume ratio of small granule; gbss, gene expression of granule-bound starch synthase; sss, gene expression of soluble starch synthase.

The correlation of physicochemical properties of wheat starch were analyzed using Pearson correlation analysis (**Figure 6**). The result showed that amylose content was positive correlation with the activity of GBSS and upregulation of *GBSS* gene, amylopectin content was positive correlation with the activity of SSS and upregulation of *SSS* gene, respectively. A significant positive correlation among the amylose content, amylopectin content, PV, TV, FV, ΔH and volume ratio of large granule, and a negative correlation among volume ratio of small granule, PV, TV, FV, ΔH were observed. The results of the analysis over the past two years are generally consistent. Therefore, combined results of GBSS and SSS enzyme activities and the gene expression together, we suggested that e[CO_2_] may promote amylose and amylopectin content by upregulating gene expression of *GBSS* and *SSS*, as well as enzyme activities of GBSS and SSS.

## 4 Discussion

Wheat, as a major global staple crop, is crucial for human survival and health. Its yield and quality play essential roles in ensuring food security and economic development (Fahad et al., 2019). The increasing atmospheric [CO_2_] potentially affects yield and quality of wheat. Previous studies by Ziska (2022) have demonstrated that within a particular range, increased [CO_2_] enhanced wheat photosynthesis and boosts its yield. Nonetheless, high concentration of CO_2_ can lead to excessive carbohydrate accumulation of wheat, potentially diminishing its quality (Wroblewitz et al., 2014). Thus, this study aims to investigate the effect of e[CO_2_] on the yield and quality of different specialized wheat cultivars, as well as the mechanism by which e[CO_2_] affects wheat starch quality, and we hope to ensure optimal wheat productivity and quality by adjusting cultivar utilization strategies under e[CO_2_] condition.

### 4.1 E[CO_2_] increases the photosynthetic rate, photoassimilate accumulation and improves yield of two specialized wheat

Chlorophyll content play a vital role in the process of photosynthesis, as it absorbs light energy and converts it into the chemical energy essential for plant growth and metabolism. SPAD value is usually used to assess plant chlorophyll content, the amount of the chlorophyll content reflects the health and growth status of the plant. In this experiment, e[CO_2_] increased the SPAD values of ZM369 and YM15 in 2021 and 2022, laying the foundation for enhanced photosynthesis. During the process of photosynthesis, CO_2_ binds with RuBisCO and undergoes a series of enzymatic reactions to participate in photosynthetic carbon fixation, ultimately producing glucose or other soluble sugars. Therefore, e[CO_2_] can affect carbon fixation and the synthesis of organic compound. Numerous studies have demonstrated that increased atmospheric [CO_2_] enhances photosynthetic efficiency of wheat (Leakey et al., 2009; Gámez et al., 2023). Leakey et al. (2009) reported that prolonged exposure to e[CO_2_] condition resulted in photosynthetic adaptation phenomenon in plant, under such condition, the vegetative organs accumulated more carbohydrates instead of grains. The accumulation of carbohydrate inhibits the expression of photosynthetic genes, resulting in a decrease in photosynthetic capacity. In our study, we didn’t observe this phenomenon, compared to a[CO_2_], e[CO_2_] enhanced the photosynthetic rate of ZM369 and YM15 during the grain-filling stage, the finding was consistent across two wheat growth seasons (**Figure 1**). These results can be partly due to variations in carbon dioxide concentration and the crop types.

The photosynthetic capacity of flag leaves is crucial for starch accumulation and grain filling. Sucrose and starch are the products of photosynthesis, however sucrose serves as a temporary storage form, while starch serves as a common storage form for photosynthetic products. The enzyme activities of SPS and SS directly impact the contents of sucrose and starch in wheat stems, leaves and grains. The plant leaves produce products (sucrose) by the process of photosynthesis, which are subsequently transferred to other parts of the plant. Enzymes including SPS, SS, GBSS, and SSS then transform these products into starch (Huang et al., 2020). Some studies indicate that elevated CO_2_ concentrations can promote the metabolism of carbohydrate, which can cause wheat leaves to upregulate the expression of SPS and SS genes, improving the production of related enzymes, and further enhancing starch synthesis (Buchner et al., 2015). In this study, e[CO_2_] upregulated the enzyme activities of SPS and SS in grains, resulting in higher starch content and lower sucrose content. This suggested that starch synthesis primarily used sucrose as a substrate, and elevated [CO_2_] promoted sucrose degradation, providing more abundant substrates for starch synthesis.

Elevated [CO_2_] affects plant photosynthesis and carbon metabolism, posing a threat to global food supply security. Similar to previous findings (Weichert et al., 2017; Gámez et al., 2023), our study found that e[CO_2_] promoted dry matter accumulation of plants, which increased grain yield of bread wheat and biscuit wheat by 17.8% and 14.3%, respectively. Furthermore, e[CO_2_] significantly increased spikelet number and thousand-grain weight for both wheat cultivars (**Supplementary Figure 3**), which was identified as the primary factor contributing to the yield improvement. It should be noted that in 2021, the dry matter weight and yield of the two cultivars were higher compared to 2022, the differences may be attribute to the varying meteorological conditions during the wheat growing seasons (**Supplementary Figure 1**).

### 4.2 The content of amylose and amylopectin are increased by upregulating gene expression and enzyme activities of GBSS and SSS under e[CO_2_]

Amylose and amylopectin are essential components of wheat grain, its quality was affected by the properties of wheat gluten, dough fermentation performance and bread volume (Li et al., 2022). Higher amylose and amylopectin contents improve ability of wheat flour to absorb water, as well as its viscoelasticity and extensibility, leading to better strength and stability of the gluten network (Wilderjans et al., 2013; Yao et al., 2020). Different cultivation conditions can change the growth environment and enzyme metabolism of wheat, thus affecting starch synthesis and accumulation, and consequently influencing the ratio of amylose/amylopectin (Li et al., 2019). Previous studies have shown that under e[CO_2_] conditions, both amylose and amylopectin contents significantly increased in wheat and other crops, the probable reason might be that the e[CO_2_] stimulated an increase in photosynthesis rate, resulting in more carbon sources being used for starch synthesis (Thompson et al., 2017). In this study, elevated [CO_2_] significantly increased the total starch, amylose, amylopectin content and ratio of two wheat cultivars, with the amylose content of ZM369 increasing by 11.06% and the amylopectin content of YM15 increasing by 4.19%, while the amylose/amylopectin of ZM369 (9.84%) was higher than YM15 (2.19%). Moreover, e[CO_2_] also regulates the activity of key enzymes involved in starch synthesis pathways such as GBSS and SSS, thus affecting the ratio of amylose and amylopectin (Xie et al., 2016). However, different specialized wheat cultivars may have varying responses to e[CO_2_].The results of our study showed that e[CO_2_] significantly increased the relative expression levels of *GBSSI*, *SSSI*, *SSSII*, and *SSSIII* genes of both wheat cultivars. Notably, the expression level of the *GBSSI* gene in ZM369 was 1.84 times higher than that of YM15 under e[CO_2_] conditions, while the expression levels of the *SSSI*, *SSSII*, and *SSSIII* genes in YM15 were 3.14, 2.97, and 1.5 times higher that of ZM369, respectively. Correlation analysis revealed that amylose and amylopectin content had positive correlation with gene expression, enzyme activity of *GBSS* and *SSS*, respectively, these results support our hypothesis.

### 4.3 Pasting properties of bread wheat YM15 is improved by influencing starch composition under e[CO_2_]

Specialized wheat cultivars are important in human diet and widely used in various flour-based food products such as bread, biscuits, noodles and pastries. The distribution of starch granules significantly affects the quality of flour-based foods. Nevertheless, starch characteristics, such as the ratio of amylose to amylopectin or granule size distribution, have often been overlooked in previous CO_2_ experiments. Starch, as a vital component of wheat, exhibits a close correlation between the distribution of starch granules and the amylose and amylopectin content. Wheat with high amylose content tends to have more small granules, while wheat with high amylopectin content tends to have more large granules (Kim and Huber, 2010). In this study, we found that under e[CO_2_] conditions, the volume ratio of large granules increased, while the volume ratio of small granules decreased in the grains of both cultivars. The changes in granule size can be explained by variations in amylose and amylopectin content. Under e[CO_2_] conditions, YM15 consistently exhibited a higher ratio of large to small granules compared to ZM369 over the two years.

The gelatinization characteristics play a crucial role in evaluating starch quality as they impact heating time, dough water absorption, and stability during food processing (Yao et al., 2020). Furthermore, the particle size distribution of starch affects the amylose and amylopectin content, as well as the gelatinization characteristics (Hong et al., 2021). Scholars have observed a negative correlation between PV, FV and the content of small granule starch (Shinde et al., 2003; Soh et al., 2006). Additionally, experiments have demonstrated that elevated [CO_2_] leads to an increase in gelatinization viscosity and a decrease in gelatinization temperature (Jing et al., 2016). However, Wang et al. (2022) found elevated [CO_2_] has no significant effect on the PV of wheat. Enthalpy represents the energy required for the gelatinization process, and it can be used along with pasting temperature to evaluate the integrity and stability of starch crystallinity. In this experiment, under e[CO_2_] conditions, the PT and ΔH of two cultivar increased significantly, indicating that the starch structural stability of the two cultivars may have been enhanced. The XRD patterns also revealed an increase in the RC of starch. Consequently, a higher gelatinization temperature is required during processing. Furthermore, when the proportion of large granule is high in starch, the values of PV, TV, FV, and BD increased during starch gelatinization (Singh et al., 2005). In this study, the increase in gelatinization enthalpy under e[CO_2_] conditions can be attributed to the higher volume ratio of large granule and the increased RC. According to Xiong et al. (2021), the amylose content significantly affects the gelatinization characteristics of starch. A high amylose content increases starch susceptibility to setback, inducing aging (Balet et al., 2019). In this experiment, ZM369 exhibited a larger increase in amylose content compared to YM15 under e[CO_2_] conditions, indicating a higher risk of aging for ZM369 starch. Additionally, studies have shown that grains with a high proportion of large granule have loosely bound starch granules and protein skeletons, making them susceptible to rupture during milling and less water-absorbent, this type of flour is suitable for making biscuit (Guan et al., 2020). In this experiment, e[CO_2_] increased the volume ratio of large granules, which may be advantageous for making biscuit. Nevertheless, further research is necessary to investigate changes in processing quality and edible characteristics of specialized wheat grown under e[CO_2_] conditions after being processed into bread and biscuits.

## 5 Conclusion

This study investigated the effect of e[CO_2_] on the starch quality of different specialized wheat cultivars and the physiological mechanisms underlying changes. Specifically, e[CO_2_] significantly improved photosynthetic performance, upregulated gene expression and enzymes activities of *GBSS* and *SSS*, increasing starch content and yield, changing starch granule distribution and pasting properties. The findings indicated that the response of two wheat cultivars to elevated [CO_2_] exhibited differences in amylose and amylopectin content, starch granule distribution, and pasting properties. These variations can be explained by the fact that ZM369 exhibited a higher upregulation of the *GBSSI* gene than YM15, whereas a lower upregulation of the *SSSI* and *SSSII* genes. The yield of two cultivars increased under e[CO_2_],

however, changes in starch characteristic and decrease in protein content contributed to the utilization value of biscuit wheat (YM15), while opposite effect on bread wheat (ZM369), which indicated that biscuit wheat is more suitable for cultivation under elevated [CO_2_]. These results provide a basis for adjusting wheat cultivar utilization strategies in response to climate change and ensuring food security.

## Data availability statement

The original contributions presented in the study are included in the article/**Supplementary Material**. Further inquiries can be directed to the corresponding authors.

## Author contributions

QW: Data curation, Formal analysis, Writing – original draft, Investigation. HP: Writing – review & editing, Investigation, Methodology, Writing – original draft. YY: Methodology, Writing – original draft. ST: Investigation, Methodology, Writing – original draft. LZ: Investigation, Methodology, Writing – original draft. HW: Investigation, Methodology, Writing – original draft. JZ: Investigation, Methodology, Writing – original draft. ZZ: Project administration, Writing – review & editing. YW: Project administration, Writing – review & editing. XW: Project administration, Writing – review & editing. XM: Funding acquisition, Project administration, Writing – review & editing. SX: Conceptualization, Funding acquisition, Writing – review & editing.
