# Supplementary material for: Effects of elevated atmospheric [CO2] on grain starch characteristics in different specialized wheat
Source: Front Plant Sci. 2024 Jan 18;14:1334053. doi: 10.3389/fpls.2023.1334053 (PMC10830628; doi:10.3389/fpls.2023.1334053)
Supplement: Supplementary file 1 [file DataSheet_1.docx]

**Supplementary Material**

**Supplementary Figures**

**Supplementary Fig. 1.** Monthly mean temperature (2021, °C; 2022, °C), monthly accumulated rainfall (mm) and CO_2_ concentration during the wheat growing season (Fer to May in 2021 and 2022) during wheat planting season.

**Supplementary Fig. 2.** CO_2_ concentration control system and modified open top chamber.

**Supplementary Fig. 3.** Dry matter weight of wheat cultivars ZM369 and YM15 under ambient and elevated [CO_2_] conditions.

**Supplementary Fig. 4.** Yield parameters of wheat cultivars ZM369 and YM15 under ambient and elevated [CO_2_] conditions.

**Supplementary Table 1.** The amylose/amylopectin ratio of cultivar ZM369 and YM15 under ambient [CO_2_] and elevated [CO_2_] conditions.

**Supplementary Table 2.** Wheat growth periods of of wheat cultivar ZM369 and YM15 under ambient [CO_2_] and elevated [CO_2_] conditions.





**Supplementary Fig. 1.** Monthly mean temperature (2021, °C; 2022, °C), monthly accumulated rainfall (mm) and CO_2_ concentration during the wheat growing season (Fer to May in 2021 and 2022) during wheat planting season.

|  |
| --- |
| **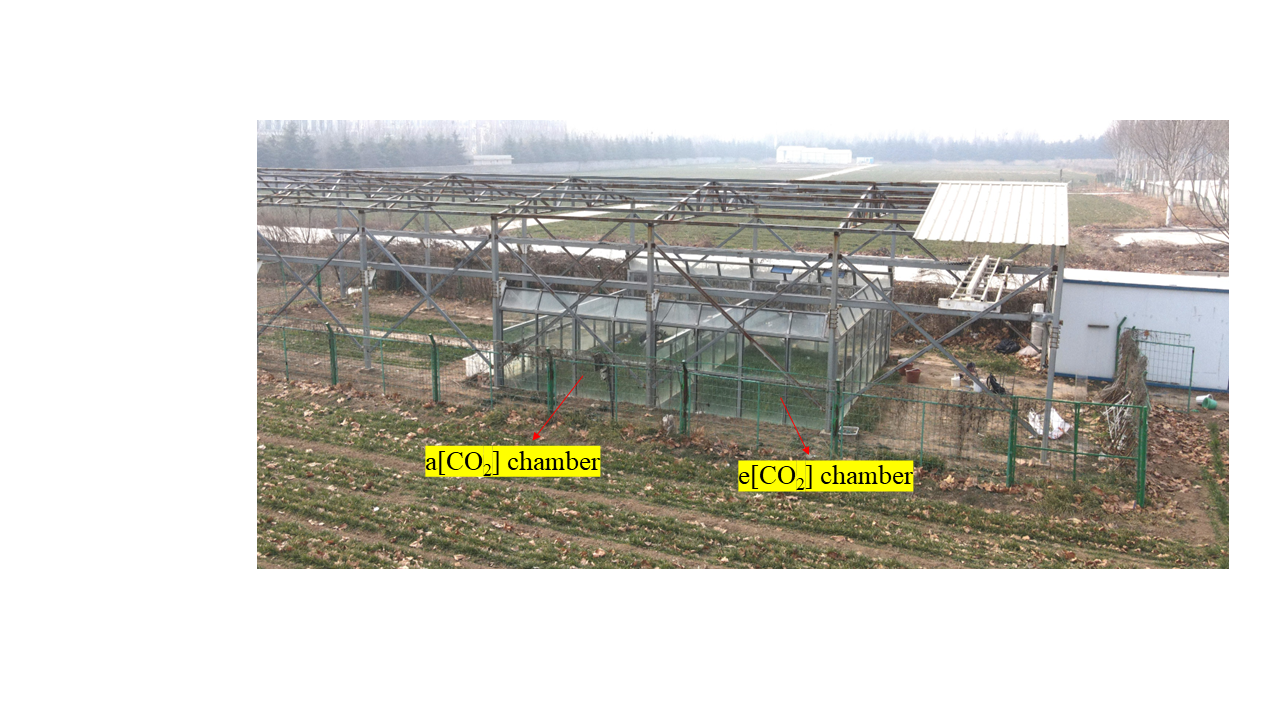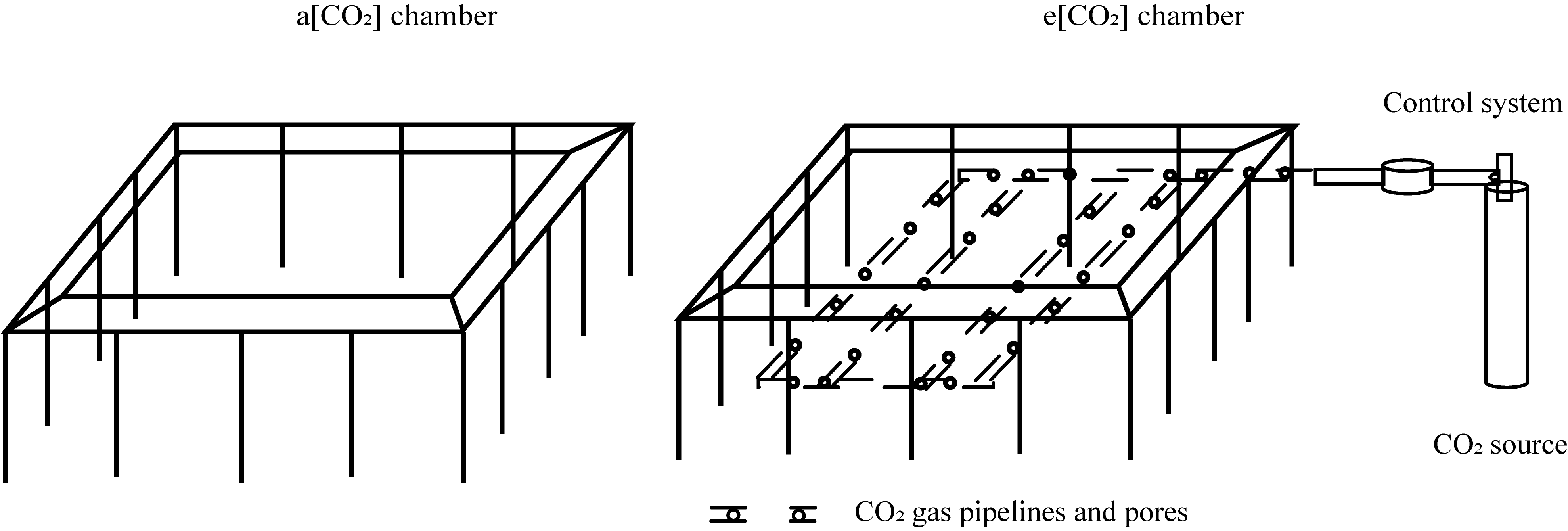** |
|  |

**Supplementary Fig. 2.** CO_2_ concentration control system and modified open top chamber.

**
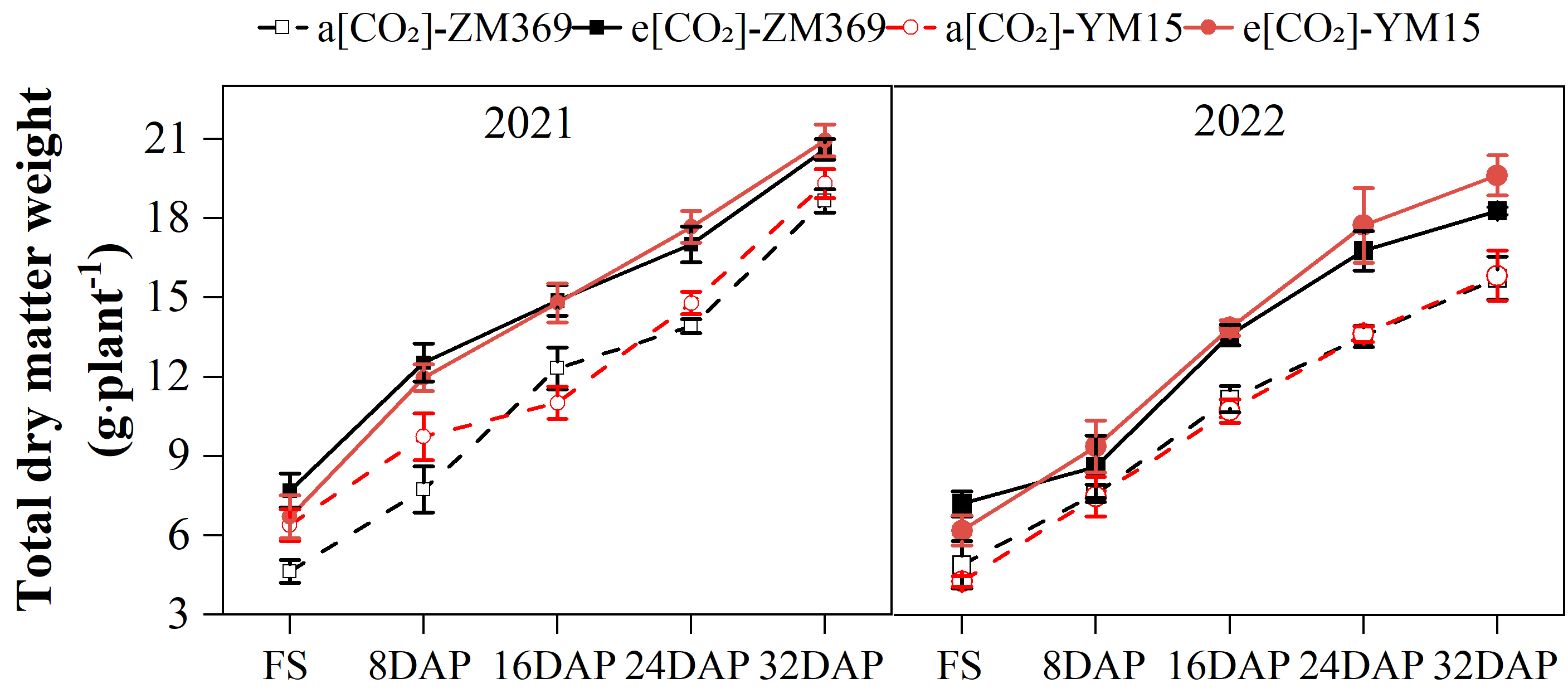
**

**Supplementary Fig. 3.** Dry matter weight of wheat cultivars ZM369 and YM15 under ambient and elevated [CO_2_] conditions. Results are presented as means ± standard deviation, and SD is indicated by error bars. Each mean has at least 3 biological replicates.

**

**

**Supplementary Fig. 4.** Yield parameters of wheat cultivars ZM369 and YM15 under ambient and elevated [CO_2_] conditions. (a) Grains per spike. (b) Spike per plant. Results are presented as means ± standard deviation, and SD is indicated by error bars. Each mean has at least 3 biological replicates. Mean values were compared using the least significance difference test (LSD). Y, year；ns, non-significant. *, Corresponds to significant differences at P ≤ 0.05; ⁎⁎, significant differences at P ≤ 0.01.

**Supplementary Table 1.** The amylose/amylopectin ratio of wheat cultivar ZM369 and YM15 under ambient [CO_2_] and elevated [CO_2_] conditions.

| Years | Cultivars | Treatment | |
| --- | --- | --- | --- |
|  |  | a[CO_2_] | e[CO_2_] |
| **2021** | ZM369 | 0.359b | 0.379a |
|  | YM15 | 0.339c | 0.341c |
| **2022** | ZM369 | 0.241c | 0.275a |
|  | YM15 | 0.264b | 0.274a |

**Supplementary Table 2.** Sampling time of wheat cultivar ZM369 and YM15 under ambient [CO_2_] and elevated [CO_2_] conditions.

| Year | Cultivar | CO_2_  treatment | JS | FS | 8 DAP | 16 DAP | 24 DAP | MS |
| --- | --- | --- | --- | --- | --- | --- | --- | --- |
| 2020-2021 | YM15 | e[CO_2_] | 17 Mar | 14 Apr | 22 Apr | 30 Apr | 7 May | 26 May |
|  |  | a[CO_2_] | 22 Mar | 18 Apr | 26 Apr | 3 May | 11 May | 30 May |
|  | ZM369 | e[CO_2_] | 20 Mar | 16 Apr | 24 Apr | 2 May | 10 May | 26 May |
|  |  | a[CO_2_] | 22 Mar | 19 Apr | 27 Apr | 5 May | 13 May | 30 May |
|  | YM15 | e[CO_2_] | 14 Mar | 8 Apr | 16 Apr | 24 Apr | 2 May | 21 May |
| 2021-2022 |  | a[CO_2_] | 20 Mar | 12 Apr | 20 Apr | 28 Apr | 5 May | 24 May |
|  | ZM369 | e[CO_2_] | 14 Mar | 8 Apr | 16 Apr | 24 Apr | 2 May | 21 May |
|  |  | a[CO_2_] | 20 Mar | 12 Apr | 20 Apr | 28 Apr | 5 May | 24 May |
